# Mechanical properties of carbyne: experiment and simulations

**DOI:** 10.1186/s11671-015-0761-2

**Published:** 2015-01-31

**Authors:** Sergiy Kotrechko, Igor Mikhailovskij, Tatjana Mazilova, Evgenij Sadanov, Andrei Timoshevskii, Nataliya Stetsenko, Yurij Matviychuk

**Affiliations:** G. V. Kurdyumov Institute for Metal Physics, National Academy of Science of Ukraine, Vernadsky Boulevard, 36, Kyiv, 03680 Ukraine; National Scientific Center, Kharkov Institute for Physics and Technology, National Academy of Science of Ukraine, Academicheskaja, 1, Kharkov, 61108 Ukraine

**Keywords:** Carbyne, Carbon monatomic chains, Strength, *Ab initio* simulation, High-field method, Field emission microscope

## Abstract

The results of the high-field technique for obtaining and testing the carbyne strength *in situ* are presented. By using molecular dynamics simulation and *ab initio* calculations, a comprehensive analysis of the results is executed. High-field technique for experimental measurement of the carbyne strength *in situ* is briefly described. It is shown that the technique used gives a lower estimation for strength of carbyne, which equals 251 GPa at *T* = 77 K. This value is close to the strength 7.85 nN (250 GPa) of contact atomic bond between carbyne and graphene sheet, from which the monatomic chain is pulled. The strength of carbyne itself is determined by strength of an edge atomic bond and it is ≈ 12.35 nN (393 GPa) at *T* = 0 K. For carbynes containing more than 10 to 12 atoms, the coefficient of elasticity (*k*_*Y*_ = 145.40 nN) and the elastic modulus (*Y* = 4631 GPa) are ascertain.

## Background

Carbon-based materials are among the most promising objects of modern nanotechnology. Carbyne is the linear allotrope of carbon. To date, carbyne, as an object of research, leaves behind graphene by the number of works, due to its unique physical, mechanical, and chemical properties [1-4], as well as due to promising applications [5-10]. The possibility of realization of these unusual functional properties is significantly depending on the strength and elasticity of carbyne. Moreover, obtaining of carbyne by unraveling of nanotubes or graphene sheets is governed by its mechanical properties [5,6,10,11]. Therefore, recently, quite a lot of works on the problem of the strength and stability of monatomic carbon chains appeared [12-17]. However, until now, the strength of carbyne was evaluated by the results of *ab initio* modeling of tension of monatomic carbon chains. Only recently, the first experimental data on *in situ* determination of the tensile strength of carbyne by high-field method have been published [18,19]. An ultra-high ultimate level of strength of these chains, which at *T* = 5 K exceeds 270 GPa, was ascertained [19]. This is more than twice higher than the experimental strength of graphene which is equal to 130 GPa [20]. Despite the considerable interest in the carbyne, information about its mechanical properties is scattered in specific works that does not allow obtaining complete representation about these properties. This work is addressed to a brief description of the experimental data obtained by measuring of strength of carbyne, as well as to the results of the molecular dynamics and *ab initio* modeling of tension of monatomic carbon chains of finite length.

## Methods

The experiments were carried out in an ultra-high-vacuum two-chamber field emission microscope (FEM) with needle-shaped specimens cooled to 4.2 and 77 K. Field ion images were formed by using helium atoms under a pressure of 10^−3^ Pa; the residual gas pressure was about 10^−7^ Pa. To use the FEM in the field and electron modes, a constant positive operating voltage of 1 to 20 kV, a varying voltage of amplitude 2 to 5 kV, and frequency of 50 Hz was supplied to carbon tips. The application of a positive voltage ensured the realization of the ion mode. The ratio of positive and negative voltages supplied to the specimen was in the interval from 8 to 14. The amplitude of negative voltage was sufficient for creating on the tip surface a field strength corresponding to an electron current in the interval 10^−9^ to 10^−6^ A. A microchannel amplifier of image brightness allowed the use of such low currents to reduce the intensity of ion bombardment of the tip surface during the flow of the field electron current in the electron mode. Carbon specimens with radii of 20 to 50 nm at the top were formed by electrochemical etching in 1 N aqueous sodium hydroxide aqueous solution with an AC voltage of 5 to 8 V from carbon polyacrylonitrile fibers. After placement in the FEM, the carbon tips were cleaned *in situ* by the methods of field desorption and by low-temperature field evaporation until a mesoscopically regular surface was formed. During this high-field forming, self-standing carbyne chains of different lengths with densities in the range of 10^15^ to 10^16^ m^−2^ are produced at the surface of the carbon specimen as a result of high-field unraveling [21,22]. Details of the experimental procedure, including *in situ* preparation of carbyne chains anchored on top of a parabolic carbon tip, are described in details elsewhere [19,21,23]. During the high-field treatment, initially sharp carbon tips were blunted up to radii of 200 to 1,000 nm. This process of formation of a ‘hairy’ carbon surface is accompanied by the destruction of a large number of carbyne chains under mechanical stresses generated by a high electric field. The field strength on the top of carbyne chains was determined using the known threshold value for the ionization of helium atoms (22 V/nm) [24]. The operation voltage was measured in these experiments with ±0.1% precision. The image magnification of the field ion microscope *M* is proportional to the ratio of the distance screen-object under study *R* and the radius of curvature of the end cap of the carbyne chain *ρ*:

1 $$ M\kern0.5em =\kern0.5em R/\beta \rho . $$

There, *β* is the geometrical image compression factor due to deviation of the shape of the specimen configuration from spherical [21]. For the paraxial trajectories of ions and electrons in a field emission microscope, the compression factor is a universal function of the ratio *r*_0_/*L*:

2 $$ \beta \kern0.5em =\kern0.5em \xi {\left({r}_0/L\right)}^{1/2}, $$

where *ξ* is a dimensionless constant equal to 1.145 ± 0.033 which is independent of the carbyne length and the tips’ radius.

For determination of the chain length *L*, the field-electron technique was applied [1]. It is based on experimental finding the compression factor *β* of field-electron images of the carbyne chains and determination of the radius *r*_0_ of the carbon tips:

3 $$ L\kern0.5em =\kern0.5em {r}_0{\left(\xi /\beta \right)}^2, $$

The surface density of ponderomotive forces at the apex of a carbyne chain was determined as:

4 $$ \sigma \kern0.5em =\kern0.5em \frac{\varepsilon_0{F}_{\mathrm{es}}}{2}, $$

where *ε*_0_ is the electric constant and *F*_es_ is the field strength on the effective electric surface at the carbyne apex at operative voltage.

Along with the experimental determination of the strength of carbyne, the work presents molecular dynamics (MD) simulation of the formation of monatomic carbon chains by pulling it out of the graphene sheet. Model of monolayers of carbon atoms included 44 of the interacting atoms and 30 boundary (edge) atoms. Boundary (edge) atoms are held in position of the lattice nodes. Such a model is a stable relatively homogeneous deformation and artifactual phase transformations. In the calculations, we used the fact that the electric forces which create axial tension are localized on top of the carbon chain. In mesoscopic electric field, induced charge is really localized at the top of a linear chain. Consequently, the mechanical strength of the electric field is localized at the top of nanowires. In our calculations, the axial mechanical load varied in the range 7 to 9 nN.

For the analysis of atomism of deformation and break of a monatomic chain, *ab initio* calculations were employed. The number of atoms in the carbyne was varied from 2 to 13. Full energies of chains were calculated by the pseudo-potential method (software *Quantum-ESPRESSO* (QE) [25]). This method was applied for modeling the mechanical properties of the chains. Pseudo-potentials for carbon were generated according to the scheme *Vanderbilt ultrasoft* using the package *Vanderbilt code version 7.3.4* [26]. To determine the accuracy of the calculation of the total energies of carbon chains, test calculations for infinite chains with the polyyne and cumulene were performed. Interatomic distances and total energies, consistent with the results of the work [27], were obtained. The value of the cutoff energy is *E*_cut_ = 450 eV.

## Results and discussion

**Figure 1:**
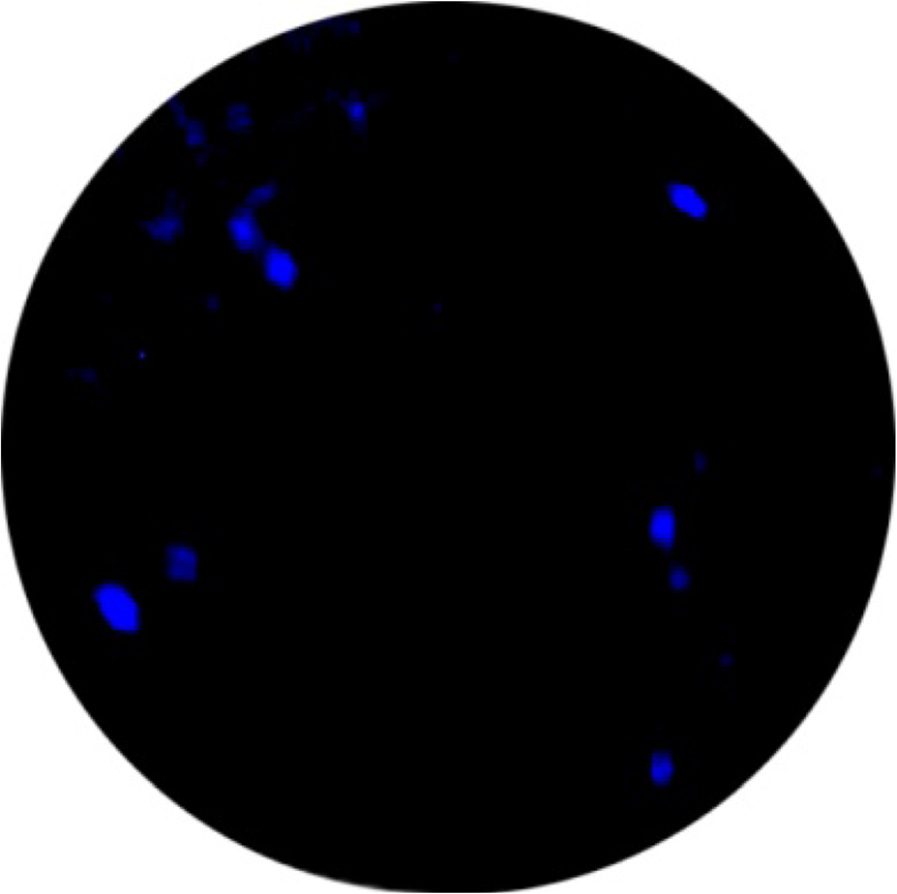
**Ion microscope image of carbyne chains at a voltage of 590 V.** The bright spots correspond to the edge atoms of carbon chains produced during a high-field treatment.

Experimental determination of the tensile strength of carbyne was executed at a temperature of 77 K. As was shown in [19], idea of the experiment is that the electric potential was raised to the critical level, at which the non-activated forming of the surface of the parabolic tip as a result of the impact of the electric field forces occurred. Formation of a ‘hairy’ carbon surface comes simultaneously with the destruction of some number of carbyne chains under mechanical stresses generated by a high electric field. At reached constant electric potential, these processes of formation and fracture of carbyne chains stop for 2 to 5 s. On lowering the electrical potential, reducing of the field strength takes place and, as a consequence, decreasing in the intensity of ionization above the tops of the chains. Below the threshold potential, the ionization process is completely stopped. At certain value of potential, close to the threshold, only a few longest carbyne chains observed as bright spots contribute to the formation of ion-microscope image (see Figure 1). In this experiment, the threshold potential was *V*_0_ = 590 V, and the maximum potential at the high-fild test reached *V*_max_ = 810 V. The ratio *V*_max_/*V*_0_ in this experiment was equal to 1.37.

Observation of such chains indicates that they survived and not destroyed at the maximum value of electric field strength, which according to Equation 4, corresponds to mechanical stress of 251 GPa. This strength value was measured at 77 K and it is 7% lower than the strength of 270 GPa previously obtained at a lower temperature 5 K [19]. Influence of local force fluctuation due to the thermal vibration of atoms in carbyne chains is one of the reasons for such difference. The distribution of carbyne chain lengths was determined by the field-electron technique using the values of compression factor and the radius *r*_0_ of the carbon tips. As the statistical analysis showed, the distribution of the carbyne chain length directly calculated from Equation 3 has a mean value of 3.5 nm, with a variance of 1.8 nm.

**Figure 2:**
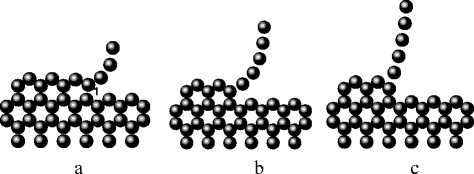
**Unraveling of monolayer graphene with formation and elongation of carbyne chains under tensile forces close to theoretical strength.** Configurations at the initial time **(a)** and after 6.4 × 10^−14^ s **(b)** and 9.6 × 10^−14^ s **(c)**.

To analyze the unraveling process, molecular dynamics simulation of the carbon chain elongation of the graphene sheet at a temperature of 77 K was carried out under conditions of constant values of the applied force. Figure 2 illustrates the process of formation of an isolated linear carbon chain under the influence of mechanical forces of the electric field by unraveling the two-dimensional atomic network on the edge of a graphene monolayer. Molecular dynamics simulation has shown that the linear chains are formed and elongated by breaking bonds of the atoms of the chain with the surface of the graphene sheet (contact atomic bond #1 in Figure 2a). Figure 2 exhibits the successive stages of unraveling the graphene under the influence of an electric field producing mechanical load 7.82 nN, resulting in elongation of the chain. Configurations at the initial time (a) and after 6.4 × 10^−14^ s (b) and 9.6 × 10^−14^ s (c) are shown.

**Figure 3:**
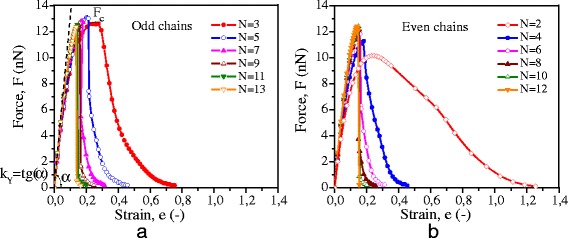
**Force** ***F*** **dependence on strain** ***e*** **for whole chain with odd (a) and even (b) number of atoms.** *F*c is the critical stresses for instability of the chains; *k*_*Y*_ is the coefficient of elasticity of the chain; *N* is the number of atoms in carbyne.

As follows from these results, the unraveling force in the interval was equal to 7.85 ± 0.05 nN and characterized strength of atomic bonds at the point of it applying to a graphene layer. If the effective diameter of the chain is 0.2 nm [23], then the value of tensile stress 250 ± 2 GPa corresponds to this value of unraveling force. This agrees well with the experimentally measured value of the critical stress 251 GPa. It means that in the experiment, the bond strength between the chain and the graphene sheet is actually measured, and the strength of the bonds within a chain should be higher. To determine this strength, *ab initio* modeling of tension of monatomic carbon chains of different lengths was executed (Figure 3).

By the results of *ab initio* calculations of chain tension, dependence of tensile force *F* on the value of total chain strain *e* was built (Figure 3). The force acting on the edge atom was calculated as:

5 $$ F\kern0.5em =\kern0.5em \frac{dE}{da}, $$

where *E* is the total electron energy and *a* is the current distances between the first and second atoms in a chain (length of edge bond).

True strain of the whole chain, *е*, was estimated as:

6 $$ e\kern0.5em =\kern0.5em 1\mathrm{n}\left(\frac{l}{l_0}\right), $$

where *l*_0_ and *l* are the initial and current chain lengths.

The values of chain strength, *F*_c_, were determined as maximum value of the force at the moment of a chain instability. Values of the coefficient of elasticity (stiffness) *k*_*Y*_ and of the elastic modulus *Y* were calculated as:

7 $$ \left.{k}_Y\kern0.5em =\kern0.5em \frac{dE}{de}\right|e=0 $$

8 $$ Y\kern0.5em =\kern0.5em \frac{k_Y}{S} $$

where *S* is the effective cross-sectional area of the chain, through which the force interaction between the atoms in the chain is transmitted. In this work, as well as in [23], the effective value of the diameter of the chain was assumed to be equal 0.2 nm.

**Figure 4:**
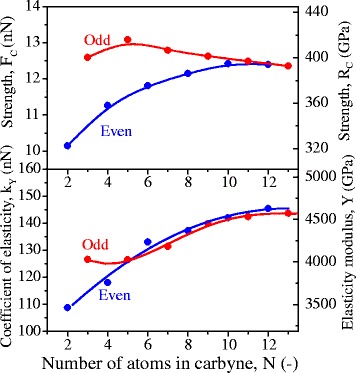
**The effect of the number of atoms in carbyne on its strength,** ***F*** **c, and elasticity,** ***k***_***Y***_ **(Y).**

According to the results of *ab initio* simulations, breaking of the chain occurs at edge atomic bond, i.e., the edge atom comes off. It means that the magnitude of strength of the whole chain is determined by the strength of edge bond. The value of this strength depends both on the total number of atoms in the chain and on whether it is odd or even number. In accordance with the obtained results, the strength of chains with an odd number of atoms is higher in comparison with the strength of ‘even’ chains. Carbyne, containing five atoms, has a maximum strength *F*_c_ = 13.09 nN. Increasing in the number of atoms in the chains gives rise to an increase in the stiffness of the chain, *k*_*Y*_. Starting from the number of atoms *N* ≥ 12, the strength becomes independent of the chain length and reaches 12.35 nN. According to the data given in [15,17], the strength of the *infinite* chain is 9.30 to 11.70 and 12.16 nN, respectively (Figure 4).

As it was noted above, the average length of monatomic chains produced at the surface of the carbon specimen is equal to 3.5 nm. A chain of such length should contain about 28 atoms. In accordance with the results of *ab initio* calculations, the strength of such a chain should be ≈ 12.35 nN (393 GPa), which is 1.46 to 1.56 times higher than experimentally measured strengths of 270 (5 K) and 251 (77 K) of the contact atomic bond between the chain and the graphene.

## Conclusions

Based on the results of our study, the following conclusions can be made:

1. Carbyne is the strongest material in the world. Experimental estimation of its strength gives the values 270 GPа (at 5 K) and 251 GPa (at 77 K). This is more than two times higher than the strength of graphene (130 GPа).
2. For carbyne of length approximately 1,36 nm, *ab initio*-calculated value equals 393 GPа (at 0 K). Such a significant difference (from 1.46 to 1.56 times) between the experimental and *ab initio* values of strength is due to the fact that in the experiment, the strength of contact bond between the chain and the graphene sheet is measured, and *ab initio* calculations gives the strength of atomic bonds in the chain itself. It means that these experimental values should be considered as a lower bound for the strength of carbyne.
3. Mechanical properties of carbyne containing more than 4 and less than 12 atoms are governed by the scale and ‘even-odd’ effects:
  ‘Even-odd’ effect manifests itself in the fact that carbynes with an odd number of atoms have the greater values of the strength.
  Scale effect lies in growth of modulus of elasticity of chain growth. The strength of ‘even’ carbynes increases monotonically, and the strength of ‘odd’ ones varies non-monotonically. Carbynes containing five atoms have the highest strength which equals 13.09 nN (417 GPa).
4. When the number of atoms in the carbyne exceeds 12, its mechanical properties become independent of the number of atoms and its parity. Strength of such carbyne is ≈ 12.35 nN (393 GPa) (at 0 K), the coefficient of elasticity - *k*_*Y*_ = 145.40 nN, and elastic modulus - *Y* = 4631 GPa.
